# Lead contamination fixation through CO_2_-based biomineralization

**DOI:** 10.1038/s41598-026-58559-y

**Published:** 2026-06-22

**Authors:** Mohammad Reza Jahanmard, Sajjad Deylaghian, Ehsan Nikooee, Hamed Aghili, Mojtaba Ansari, Ehsan Khodayari, Ali Niazi

**Affiliations:** 1 Department of Civil and Environmental Engineering, Shiraz University, Shiraz, Iran; 2 Department of Materials Science and Engineering, Shiraz University, Shiraz, Iran; 3 Institute of Biotechnology, Shiraz University, Shiraz, Iran

**Keywords:** Lead contamination, CO_2_ biological sequestration, MICP, Carbonic anhydrase, *Sporosarcina pasteurii*, Heavy metal, Biogeochemistry, Biotechnology, Chemistry, Environmental sciences, Microbiology

## Abstract

Ureolytic microbially-induced carbonate precipitation and ureolytic enzymatically-induced carbonate precipitation have been proposed in recent years as emerging environmentally friendly techniques for heavy metal fixation. However, they rely on the hydrolysis of urea for generating carbonate ions needed to immobilize heavy metal cations. In this study, for the first time, a novel alternative is proposed, and its feasibility for lead removal in aqueous solutions is examined. The method relies on direct CO_2_ biocementation. Where contaminated solutions are exposed to CO_2_, and CO_2_ hydration and formation of carbonate ions is biologically facilitated through (I) microbial strains capable of producing carbonic anhydrase enzyme (i.e., microbially-facilitated carbonation, MFC), and (II) an enzymatic solution containing bovine CA enzyme (i.e., enzymatically-facilitated carbonation, EFC). The results of atomic absorption spectrophotometry indicate up to 99% Pb removal achieved in both MFC and EFC, comparable to the control ureolytic test (98%). However, the former (proposed) techniques are ammonium-free and more environmentally benign. Furthermore, they provide a dual environmental benefit: a method for carbon capture through biomineralization and its utilization for heavy metal removal. The use of Tris buffer considerably enhanced the efficiency of enzymatically-facilitated carbonation for Pb removal (i.e., increasing it from 78% to 99%). The results of microfabric analysis of precipitates (i.e., SEM imaging, XRD, FTIR, and Raman spectroscopy) confirmed the formation of calcium and lead carbonates such as calcite, cerussite, and hydrocerussite, indicating potential removal mechanisms such as lead carbonate precipitation as well as Pb trapping inside calcium carbonate crystals.

**Supplementary Information:**

The online version contains supplementary material available at https://doi.org/10.1038/s41598-026-58559-y.

## Introduction

Rapid industrialization has led to the uncontrolled release of heavy metals into the environment; consequently, heavy metal pollution has become a rising global concern^1,2^. The term “heavy metals” refers to metals with atomic weights between 63.5 and 200.6 and densities greater than 4.5 g/cm^3^^3,4^. Several agricultural, petrochemical, and industrial sources have contributed to heavy metal contamination of land and water worldwide. These include mine tailings, e-waste discharge, leaded gasoline and paints, animal manure, leather tanning, sewage sludge, wastewater irrigation, coal combustion, smelting industry, fertilizers, and pesticides^5–14^. Future food insecurity is aggravated by heavy metals as they reduce crop growth and yield, and restrict available agricultural land^15,16^. Due to the non-biodegradability and bioaccumulation tendency of heavy metals, their presence in soils and water can lead to their transfer and accumulation in humans’ bodies through the food chain^15,17–20^. Therefore, heavy metals require immediate action as they pose serious threats to human well-being and Earth’s ecosystem health.

One of the most widely used heavy metals is lead (Pb), discovered and utilized by humans for millennia^21^. The oldest Pb archaeological relics found belong to 6400 BC^22,23^. Pre-Incan cultures, Tiwanaku and Wari, living in the western present-day Peru, also utilized lead for silver purifying and extraction^24^. In the Roman Empire, lead acetate was used as a beverage sweetener. It is estimated that Romans might have had daily lead consumption as much as a gram^25^. Since the industrial revolution, and in the modern era, lead has been used in car batteries, coating industrial tanks, in smelting, and as a pigment in plastics^25–27^. It can also be released to the environment as a result of lead emissions from petrol, acid mine drainage (AMD), as well as Pb-leakage from perovskite solar cells^25,28–31^. Table 1 reports Pb concentration in various aquatic environments and anthropogenic waste sources.

What makes Pb contamination concerning is not only its prevalence in the built environment but also its toxicity. It is the second most toxic metal on Earth^32,33^. It appears, after arsenic, as the second most significant potential threat to human health by the Agency for Toxic Substances and Disease Registry (ATSDR)^34^. The World Health Organization estimates that 2 million lives and 53 million disability-adjusted life-years were lost in 2019 due to exposures to selected chemicals. Nearly half of the deaths attributable to chemical exposures in 2019 were due to lead exposure and resulting cardiovascular diseases^35^. Lead exposure and poisoning can result in several health issues, including nerve damage and behavioral problems, reproductive disabilities, high blood pressure, kidney failure, cardiovascular disorders, and anemia. Furthermore, lead has been known to be carcinogenic. A less developed blood-brain barrier in children makes them susceptible to adverse effects of lead, such as Intelligent Quotient (IQ) deficiency, behavioral disorders, and brain development impairment^36–38^. Due to several adverse effects of Pb on human health, it has been considered as a “chemical of very high concern”^16,39^.

**Table 1:** Pb concentration in various waste sources and aquatic environments.

Row no.	Source type/example		Typical lead concentration range	Ref.
1	Irrigation wastewater		0.0196 mgL^− 1^ to 52.4 mg L^− 1^	Ali et al.^40^
2	Contaminated groundwater	Untreated/heavily contaminated groundwater (e.g., Abakaliki, southeast Nigeria)	0–38 mgL^− 1^	Obasi and Akudinobi^41^
3	Industrial wastewater	Waste streams from electroplating, anodizing, and metal finishing	0.5 to 100 mgL^− 1^	Gui and Blackwood^42^ Metwally et al.^43^
4		Battery manufacturing and recycling	1.0 to 10.0 mgL^− 1^, potentially reaching up to 50.0 mgL^− 1^ in untreated effluents	Metwally et al.^43^
5		Mining and smelting	0.1 to 20.0 mgL^− 1^	
6		Paint and pigment manufacturing	0.5 and 15.0 mgL^− 1^	
7		Electronics manufacturing	0.1 to 3.0 mgL^− 1^	
8		Glass and ceramics production	0.2 to 5.0 mgL^− 1^	
9		Textile and dyeing industries	0.1 to 2.0 mgL^− 1^	
10		Petroleum refining	0.05 to 2.0 mgL^− 1^	
11		Automotive and aerospace industries	0.1 to 4.0 mgL^− 1^	
12		Chemical manufacturing	0.5 to 8.0 mgL^− 1^	
13		Pesticide and fertilizer production	0.1 to 5.0 mgL^− 1^	
14	Typical municipal solid waste (MSW) landfill leachate		0.65 to 2.17 mgL^− 1^	Beinabaj et al.^44^

Various techniques, including chemical, physical, or a combination of them, are commonly employed to remediate water and soil contaminated with heavy metals^45,46^. These methods generally exhibit limitations, including inadequate efficiency and elevated costs^47^. As an alternative solution and inspired by natural processes, biomaterials and biological processes have recently been employed in various hydrogeological, environmental, and geotechnical engineering purposes, from controlling seawater intrusion, water and wind erosion, and construction of bio barriers to stabilization of problematic soils^48–64^. The use of biological methods for Pb removal has also been proposed in recent years. These methods harvest the metabolic paths of microorganisms and their associated biochemical reactions. Microbially induced carbonate precipitation (MICP) is one such method that utilizes the microbially induced precipitation of calcium carbonate as an environmentally friendly technique to remove heavy metals, particularly lead and cadmium^65–73^. However, conventional microbially-induced carbonate precipitation relies on the urease enzyme and hydrolysis of urea (Eqs. 1–5) to produce carbonate ions required for Pb fixation through its precipitation. Urea hydrolysis results in the formation of ammonium ions^74^. Production of ammonium ions in large amounts can contribute to groundwater contamination and would also alter soil pH, affecting the soil’s indigenous ecosystem.

1 $$\:{CO\left({NH}_{2}\right)}_{2}+2{H}_{2}O\;\underrightarrow{urease}\;{2NH}_{3}+{H}_{2}C{O}_{3}$$

2 $$\:{NH}_{3}+{H}_{2}O\to\:{NH}_{4}^{+}+{OH}^{-}$$

3 $$\:{H}_{2}C{O}_{3}\leftrightarrow\:{CO}_{3}^{2-}+2{H}^{+}$$

4 $$\:{Ca}^{2+}+{CO}_{3}^{2-}\to\:{CaCO}_{3}\downarrow\:$$

5 $$\:Metal\:\left({M}^{2+}\right)+{CO}_{3}^{2-}\to\:{MCO}_{3}\downarrow\:$$

Global CO_2_ emissions have surpassed 41 billion tonnes in 2024^75^. The surge in the atmospheric levels of carbon dioxide (CO_2_) is another major environmental challenge, given its key role in global warming^76^. It is, therefore, crucial to develop not only low-carbon and environmentally-friendly technologies but also negative emission techniques. The direct CO_2_ utilization seems crucial more than ever. The direct CO_2_ mineral trapping can provide a method to remove heavy metals while serving the second purpose, which is the carbon utilization by conversion of gaseous CO_2_ to minerals. This process is, however, limited by the inherently slow kinetics (rate constant, k ~ 0.04 s^− 1^) of CO_2_ hydration (Eq. 6)^77–79^.

6 $$CO_{2(g)}\leftrightarrow CO_{2(aq)}+H_{2}O\leftrightarrow H^{+}+HCO_{3}^{-}\leftrightarrow 2H^{+}+CO_{3}^{2-}$$

In biological systems, the hydration of CO_2_ is catalyzed by carbonic anhydrase (CA)^80^, with a rate constant as high as 10^6^ s^− 1^. Therefore, one natural alternative would be to accelerate the carbonation process and target both heavy metal removal and CO_2_ sequestration via carbonic anhydrase (CA)-facilitated carbonation of contaminated samples. For this purpose, both cell-free enzymes and microorganisms, such as *Sporosarcina pasteurii*, can be employed. Carbonic anhydrase enzyme (CA) is encoded in the genome of *S. Pasteurii* (i.e., γ-CA and β-CA with WP_115362601 and WP_115363014 NCBI Reference Sequence numbers, respectively^81,82^. In the presence of a calcium source or calcium- containing geomaterials, the hydrated CO_2_ reacts with available calcium cations and forms calcium carbonate (Eq. 8). Therefore, Pb can be contained and trapped within the precipitated calcium carbonate particles, but it can also enter direct reaction with the carbonate ions formed during hydration of gaseous CO_2_ (Eq. 9).

7 $$\:{\mathrm{C}\mathrm{O}}_{2}+{\mathrm{H}}_{2}\mathrm{O}\;\underrightarrow{\mathrm{C}\mathrm{A}}\;{\mathrm{H}\mathrm{C}\mathrm{O}}_{3}^{-}+{\mathrm{H}}^{+}$$

8 $$\:{Ca}^{2+}+{\mathrm{H}\mathrm{C}\mathrm{O}}_{3}^{-}\to\:{CaCO}_{3}\downarrow\:+{\mathrm{H}}^{+}$$

9 $$\:Metal\:\left({M}^{2+}\right)+{CO}_{3}^{2-}\to\:{MCO}_{3}\downarrow\:$$

Although solution chemistry of carbonate ions precipitating divalent metals has been known since the mid-1800’s and using CA to accelerate the reaction between CO_2_ and H_2_O for carbonate precipitation has been studied in the existing literature, the novel aspect of the current study is to employ this approach for heavy metal fixation. To the authors’ knowledge, the use of carbonic anhydrase-facilitated carbonation (and CA-assisted biomineralization using direct CO_2_ gas supply) for heavy metal remediation has received limited attention in the literature. Particularly, the performance of microbially-facilitated carbonation (MFC) and enzymatically-facilitated carbonation (EFC) (through CA enzyme) for heavy metal remediation has not been compared. In addition, there is no systematic study examining the precipitated calcium and lead carbonates through MFC, EFC, and ureolytic MICP, providing insights into mechanisms behind lead removal in these techniques. The current study aims to address these research gaps.

In this study, the lead-contaminated water samples were subjected to CA-facilitated carbonation by direct use of cell-free CA enzyme as well as by CA-producing *S. pasteurii* to examine the potential and feasibility of lead removal through direct CO_2_ sequestration (i.e., in MFC, and EFC, the contaminated aqueous solutions were exposed to CO_2_ gas and no urea was supplied). Furthermore, the study is accompanied by a thorough analysis of produced precipitates to elucidate the mechanisms behind lead removal through microbially- and enzymatically-facilitated CO_2_-induced biomineral trapping, comparing them with conventional ureolytic MICP. It should be noted that MFC and EFC provide a negative emission approach as compared to conventional biological methods of heavy metal removal, such as conventional MICP and EICP. Besides, MFC and EFC do not result in ammonium ions, which bring environmental concerns, and are common by-products of conventional ureolytic EICP and MICP. Hence, the proposed approach would not only be ammonium-free but also provide the possibility of direct carbon capture as EFC and MFC benefit from a direct CO_2_ gas supply rather than urea hydrolysis.

## Materials and methods

### Materials

*Sporosarcina pasteurii* (strain designation PTCC 1645, or ATCC 11859), culture medium of Nutrient Broth (Sigma-Aldrich^®^), calcium chloride (Merck, CAS:10035-04-8), Carbonic Anhydrase (CA) from bovine (EC 4.2.1.1) (Creative Enzyme), urea (for control urease-based tests) (Sigma-Aldrich, catalog number 51456), Pb(NO_3_)_2_ (Chem Lab NV: CAS: 10099-74-8), Tris(Hydroxymethyl)aminomethane (Alfa Aesar, CAS: 77–86-1) also known as Tris Buffer and carbon dioxide gas (for experiments based on direct CO_2_ utilization) were used in this study. All solutions were prepared using deionized water.

### Preparation of the enzymatic solution

Carbonic anhydrase (CA) catalyzes the hydration of carbon dioxide (CO_2_) in an aqueous environment into bicarbonate (HCO_3_^−^) and protons (H^+^), which is a very slow process in its absence. The zinc ion at the active site of CAs plays a key role in catalyzing this reaction. This study utilized the α-class carbonic anhydrase from bovine erythrocytes (CA II) due to its high catalytic efficiency, structural stability, and wealth of studies on its chemical characteristics, making it a suitable candidate for the enzymatic pathway. To prepare the enzymatic solution, a solution of 3 mg/L of CA enzyme (i.e., with equivalent activity of 9 U/mL of the solution) was employed, which has proved to be adequate to trigger the biomineralization processes in the previous studies^83^.

### Preparation of bacterial solution

*Sporosarcina pasteurii* strains, producing β and γ-class carbonic anhydrases, were selected to examine the potential of microbially-facilitated carbonation to remove heavy metals. The culture medium required for bacterial cultivation was prepared by adding 25 g of nutrient broth to deionized water. To sterilize, it was autoclaved for 30 min at 121 °C at a pressure of 15 psi. After adding a single colony of the *Sporosarcina pasteurii*, the prepared bacterial solution was incubated using a shaker incubator at a rotating speed of 200 rpm and a temperature of 30 °C for 24 h. The concentration of bacteria in the culture medium was monitored by measuring the optical density (OD) at a wavelength of 600 nm using a spectrophotometer (UV-2120 Optizen, Mecasys) until the bacteria reached the desired OD of 1.6, The preparation procedure complies with previous studies to ease the comparison of results^59,84^.

### Bioremediation experiments

The enzymatic and microbial pathways for calcium carbonate (CaCO_3_) precipitation were tested using a CO_2_ diffusion system (i.e., a gas chamber). The enzyme and bacterial solutions were mixed following the experimental design scenarios explained hereafter. In the urease-based control tests (where, instead of direct use of CO_2_, hydrolysis of urea was employed to generate carbonate ions), urea was added to the solution in the same molarity as the calcium source. It is noteworthy that in the presence of urea in the environment, urease enzymes of *Sporosarcina pasteurii* strains are triggered and catalyze the urea hydrolysis, thereby generating carbonate ions. Whereas in the absence of urea, and by exposing the solution to CO_2_ in a gas chamber, the CA enzymes of bacterial strains facilitate the hydration of gaseous CO_2_ (previously explained in Eqs. 6 and 7, resulting in CaCO_3_, and PbCO_3_ precipitation and thus heavy metal remediation).

In order to compare the Pb removal performance of CO_2_-driven biomineralization versus urea-based microbial carbonate precipitation, different test scenarios were considered. As biomineralization in both techniques relies on the reaction of carbonate ions, obtained either from hydration of gaseous CO_2_, facilitated through cell-free CA enzyme (or bacterial CA), or carbonate ions generated from hydrolysis of urea, three series of experiments were considered: one through the bacterial urease pathway, and the other two through direct CO_2_ utilization (bacterial and enzymatic pathways). Furthermore, control tests were considered; they were control tests with and without a calcium source (i.e., CaCl_2_ solution), where the contaminated solution was exposed to CO_2_ in the chamber but in the absence of bacterial and enzymatic solutions. Detailed explanation of these test scenarios is as follows.

#### Control urease-based test series (MICP through bacterial urea hydrolysis)

To examine the efficiency of direct CO_2_ utilization as compared to the conventional MICP technique utilizing hydrolysis of urea, these experiments were considered. To prepare samples, a solution with a volume of 20 cc containing 0.1 M CaCl_2_, 0.1 M urea, and 0.5 mM Pb(NO_3_)_2_ was filtered through a filter with a constriction size of 0.45 micrometer. 5 cc of a bacterial solution with an OD of 1.6 was added to the sample. The samples were kept at room temperature for 7 days. Finally, 10 cc of each sample was filtered by a filter with a porosity of 0.45 micrometers, and their lead concentration was measured by an atomic absorption spectrophotometer. The Pb concentration of 0.5 mM (≈ 100 ppm, ≈ 106 mg L^− 1^) was selected to account for a high Pb concentration representing real-world applications, among those in Table 1 (i.e., cases 1–4, particularly 4), presented earlier in the introduction.

#### Direct CO_2_ utilization (MICP through microbially-facilitated carbonation (MFC))

To prepare samples, a solution with a volume of 20 cc with a concentration of 0.1 M CaCl_2_ and 0.5 mM Pb(NO_3_)_2_ was filtered through a filter (opening size: 0.45 micrometer). 5 cc of a bacterial solution with an OD of 1.6 was added to the sample. Previous studies indicate that exposure to CO_2_ tends to reduce solution pH and hinder mineralization efficiency^83^. Therefore, prior to exposure to CO_2_, and in order to investigate the effect of Tris on the performance of the method, samples were prepared with and without Tris. In samples with Tris, Tris in dried powder form was added to samples (with a volume of 20 cc) to reach a concentration of 0.1 mM prior to the addition of bacterial solution (5 cc). It has been proposed that Tris buffer (Tris (Hydroxymethyl)aminomethane) can enhance CO_2_ solubility trapping by controlling pH during CO_2_ hydration^85^. However, the effect of Tris Buffer on CO_2_-based heavy metal trapping (i.e., heavy metal fixation through enzymatically and microbially-facilitated carbonation) has not been explored.

The samples were incubated at room temperature in a CO_2_ chamber with an average relative pressure of 1 bar for 7 days. Finally, 10 cc of each sample was filtered through a filter (with a constriction size of 0.45 micrometers), and its lead concentration was measured by an atomic absorption spectrophotometer. Measurements were made in triplicate to ensure the reproducibility of the results.

#### Direct CO_2_ utilization (MICP through enzymatically-facilitated carbonation (EFC))

For the preparation of samples in this test scenario, 20 mL solutions were prepared containing 0.1 M CaCl_2_ and 0.5 mM Pb(NO_3_)_2_. These solutions were filtered through a membrane filter with a pore size of 0.45 micrometers to ensure clarity and remove particulates. To evaluate the effect of Tris on the performance of the method, samples were prepared both in the presence and absence of Tris. For samples containing Tris, a dried Tris powder was added to the 20 mL solution to achieve a final concentration of 0.1 mM prior to the addition of the enzyme. Then, 5 cc of a CA enzyme solution with a concentration of 3 mg/L was added to the sample. The samples were incubated at room temperature in a CO_2_ chamber with an average relative pressure of 1 bar for 7 days. Finally, 10 cc of each sample was filtered by a filter (opening size: 0.45 micrometer), and its lead concentration was measured by an atomic absorption spectrophotometer.

#### Control carbonation sample (exposure to CO_2_ without enzymatic and bacterial solutions)

To prepare the required samples, a solution with a volume of 20 cc with a concentration of 0.5 mM Pb(NO_3_)_2_ was filtered through a filter (opening size: 0.45 micrometer). 5 cc of deionized water was added to the sample. In another control series, a solution with a volume of 20 cc with a concentration of 0.1 M CaCl_2_ and 0.5 mM Pb(NO_3_)_2_ was filtered through a filter (with 0.45 micrometer-size opening), followed by the addition of 5 cc of deionized water. The control samples were exposed to CO_2_ at room temperature in a CO_2_ chamber with an average relative pressure of 1 bar for 7 days. Finally, 10 cc of the sample was filtered through a filter with a constriction size of 0.45 micrometers, and its lead concentration was measured by an atomic absorption spectrophotometer.

### Microfabric and physicochemical analysis

Microfabric analysis of the precipitates resulting from the treatment of contaminated solutions with enzymatically and microbially facilitated carbonation provides insights into the mechanisms of lead removal through these processes. For this purpose, we utilized Raman spectroscopy, scanning electron microscopy (SEM), X-ray Diffraction (XRD), and Fourier Transform Infrared Spectroscopy (FTIR) to characterize the precipitates, verify the presence of Pb in the precipitates, and identify the type and polymorph of precipitated crystals. The Raman spectroscopy was conducted using a Lab Ram HR confocal Raman spectrometer (Japan) employing a 523 nm laser wavelength. The SEM was conducted using a TESCAN VEGA3 instrument (TESCAN, Czech Republic) equipped with Energy Dispersive Spectroscopy (EDS). The samples were coated with a 5 nm layer of gold prior to testing. The XRD was performed using a D8 ADVANCE equipped with Cu kα radiation (λ= 1.542 Å). The data were collected over a 2θ range of 10° to 70°. Phase determination and quantitative phase estimation were performed using the Rietveld-based refinement implemented in X’Pert HighScore Plus software. The obtained phase fractions are reported as approximate values. FTIR analyses were carried out using a Tensor II (Bruker, Germany).

## Results and discussion

### Results of Pb removal efficiency

**Fig. 1:**
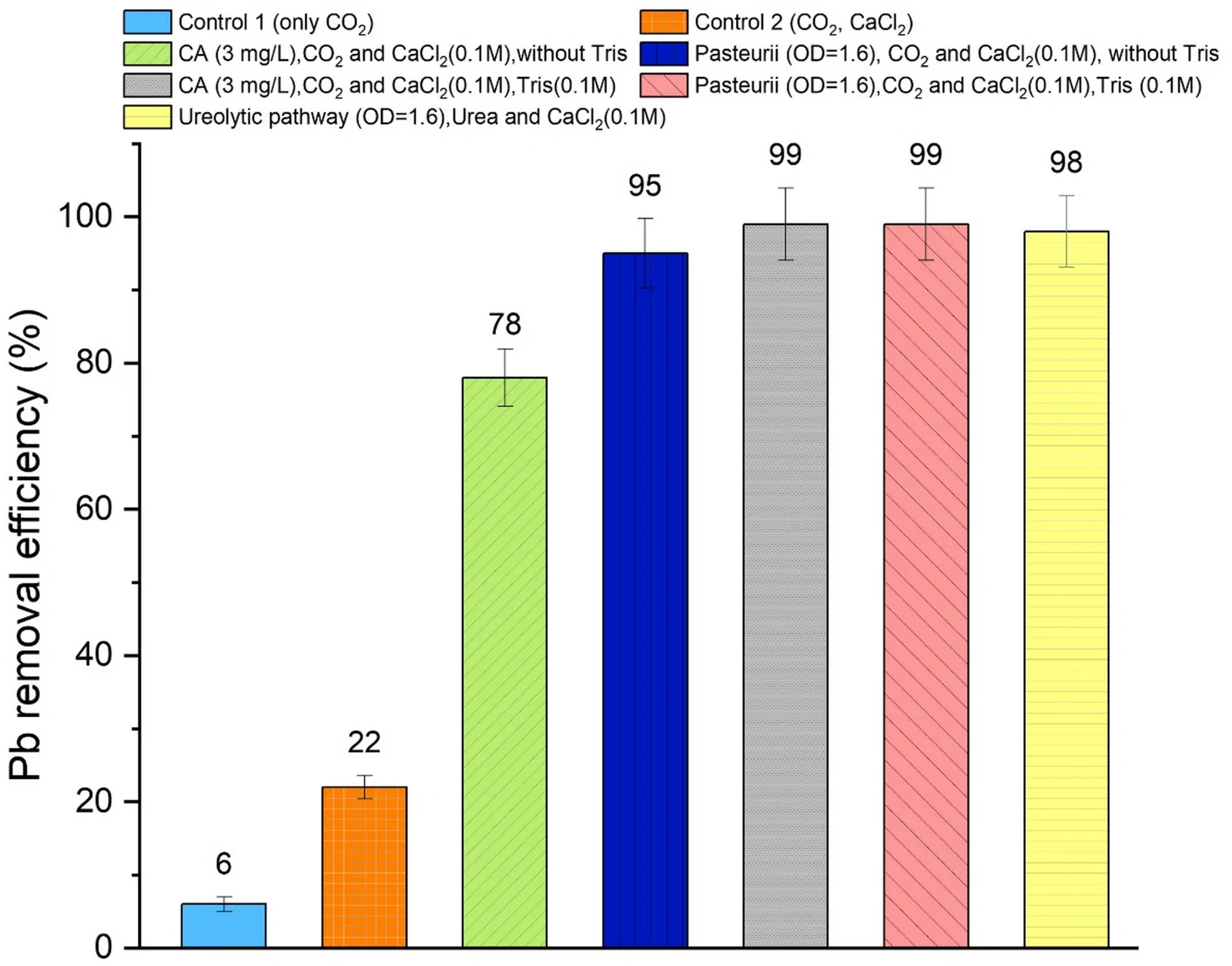
presents the potential of different microbial and enzymatic pathways examined in this study for Pb removal (F-value = 299.2, P-value < 0.0001).

Figure 1 compares the effect of enzymatically-facilitated carbonation, microbially induced carbonation and ureolytic biomineralization pathways on Pb removal. As can be seen, the addition of bacterial and enzymatic solutions has increased Pb removal efficiency considerably as compared to control samples (i.e., those samples, which do not include enzyme and bacterial solution), from 4% (for CO_2_ exposure only) and 22% (cementation solution only+CO_2_ exposure), to 78%, for enzymatic pathway and to 95% for bacterially-facilitated carbonation. The addition of Tris buffer, which helps to stabilize pH and facilitates the mineralization process, increases the Pb removal efficiency even further and leads to a 21% increase in the efficiency of enzymatic pathways and a 4% increase in the efficiency of microbially induced carbonation.

**Fig. 2:**
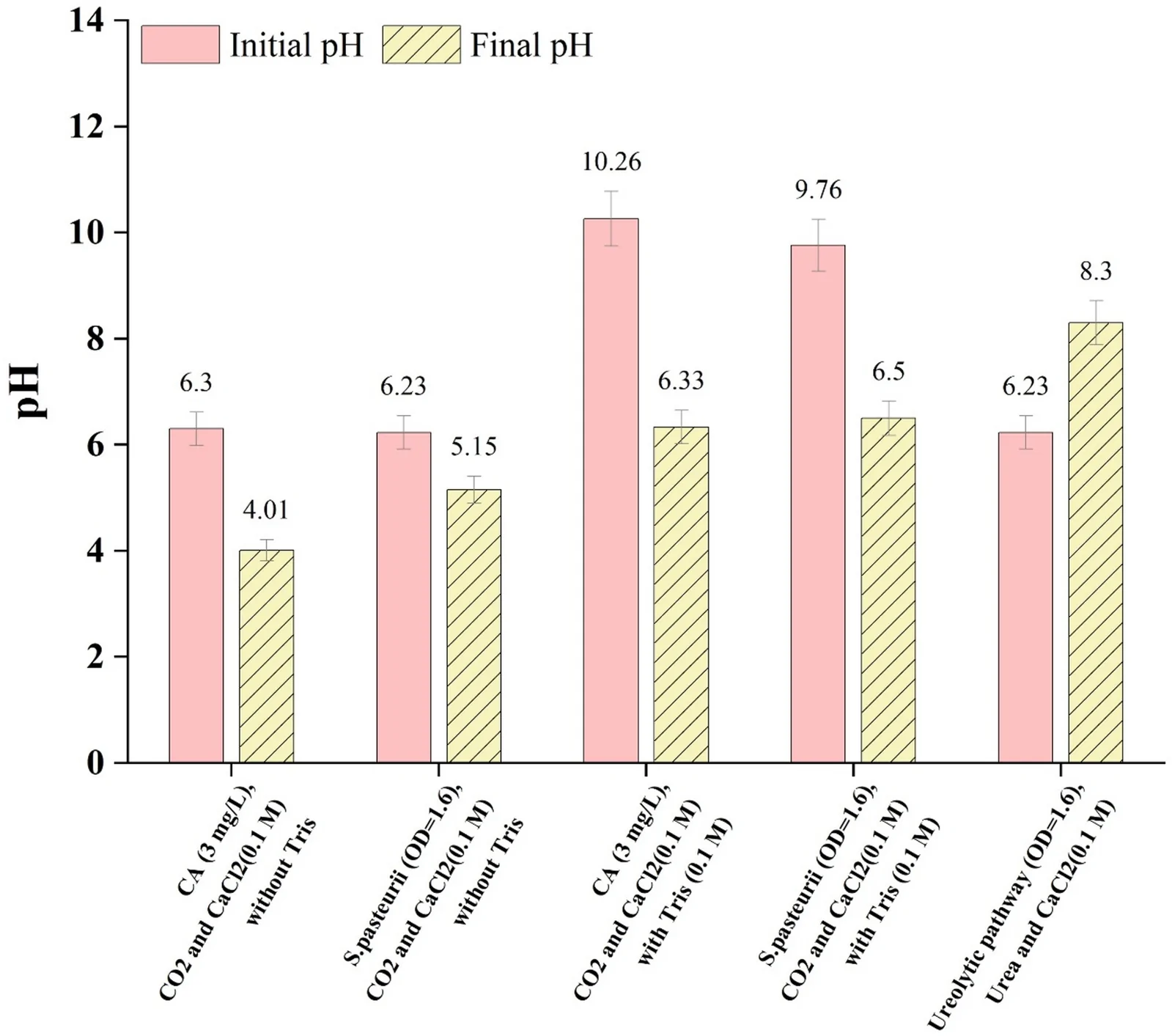
Initial and final pH values of the experimental groups (F-value = 80.5, 79.8, respectively, P-value < 0.0001 for both).

The effect of Tris Buffer on the results has also been further examined in light of pH changes and the pH sensitivity of the CA enzyme. Figure 2 presents the initial and final pH of solutions obtained using different techniques, namely, enzymatically facilitated carbonation (EFC), microbially facilitated carbonation (MFC) in the presence and absence of Tris Buffer, and ureolytic microbially induced carbonate precipitation (Ureolytic MICP). The drastic enhancement of lead removal by Tris buffer in the cell‑free bovine carbonic anhydrase (CA) system, compared to the less pronounced improvement in the bacterial CA system, is explained by the distinct pH‑CO_2_ hydration activity profiles of the different CA classes. Bovine α‑CA has been reported to be pH-sensitive in previous studies, and its activity drops significantly at low pH values^86^. In the Tris unbuffered case (pH = 6.3), the α‑CA CO_2_ hydration activity (coefficient) at start is already low (i.e., 12.5% of its maximum value, ~ 7.9 × 10⁴ s^−1^). At a pH of 4.01 (i.e., after the treatment period), the enzyme exhibits an extremely low hydration potential (i.e., only 0.1% of its maximum). Adding Tris Buffer results in a high initial pH of 10.26 for the buffered enzymatic solution, where the enzyme experiences its maximum hydration coefficient (~ 6.3 × 10^5^ s^−1^). At the end of the removal process, the pH drops to pH 6.33, and the enzyme has still retained part of its activity, around 13.2% of its CO_2_ hydration potential (i.e., ~ 8.3 × 10⁴ s^−1^). *Sporosarcina pasteurii*, however, produces β‑Ca, and γ‑CA enzymes; they are encoded in its genome (i.e., γ-CA and β-CA with WP_115362601 and WP_115363014 NCBI Reference Sequence numbers, respectively^81,82^. Other groups of CA-enzymes, namely, γ‑CA and β-CA type I, are less sensitive to pH change, and retain a higher portion of their activity (i.e., CO_2_ hydration potential) as pH drops, compared to α‑CA^87,88^ (i.e., ~ 30 to 50% of their maximal activity at pH of 6.2 compared to Bovine α‑CA, which retained only 12.5% of its maximum activity at pH of 6.3). Furthermore, the bacterial cell wall and periplasm act as a microenvironment, buffering pH locally and bringing further protection for the bacterial CA enzyme against a harsh environment. This explains why Tris Buffer addition has provided less enhancement in lead removal through bacterially facilitated carbonation than enzymatically facilitated carbonation and biomineralisation (i.e., through Bovine α‑CA cell-free enzyme, where the addition of Tris Buffer has drastically enhanced the enzyme’s performance). It is noteworthy that the absence of reaction kinetics curves and leaching/long-term stability tests is an important challenge and limitation to further validate and understand the mechanisms behind the observed removal mechanisms. Given the design of the current study (the use of a sealed box for CO_2_ exposure), no direct access to samples was possible to measure pH and analyze the kinetics of reactions during the treatment. This remains a significant limitation, which is suggested to be addressed in future studies.

It is notable that microbially induced calcium carbonate precipitation via the ureolytic pathway, which requires urea addition, has also achieved almost the same efficiency in Pb removal. However, the biomineralization through microbially-facilitated carbonation (CA-enzyme generated by *S. pasteurii*) and enzymatically-facilitated carbonation (i.e., through cell-free CA-enzyme) provides two important advantages:

1. The ureolytic pathway for biomineralization requires the addition of urea, where the decomposition of urea and its hydrolysis produce carbonate ions, and thereby, the formation of calcium carbonate minerals as well as lead carbonate minerals, and eventually, Pb biomineral trapping and removal. However, ammonium ions are formed in this process, which remain in the environment and are not environmentally desirable, most notably when they are produced in large volumes. Such unfavorable by-products are not produced during biomineralization through enzymatically and microbially facilitated carbonation by means of CA enzyme or CA-producing bacteria, in our case, namely, *Sporosarcina pasteurii*. In the latter two biomineralization pathways, the CO_2_ gas hydration is facilitated using the CA-enzyme. The produced carbonate ions react with Pb and Ca cations present in the medium and are converted to biominerals trapping Pb.
2. The bacterially and enzymatically facilitated carbonation provides a method to capture and utilize CO_2_ without the need for the addition of any extra substance (e.g., urea), reducing the CO_2_ footprint (and even sequestering it through biomineralization).

In order to further investigate the crystals formed in the precipitates separated from the solution, different microfabric analyses are performed, the results of which are presented in the subsequent sections.

### Microfabric analysis of precipitates

#### Results of SEM

**Fig. 3:**
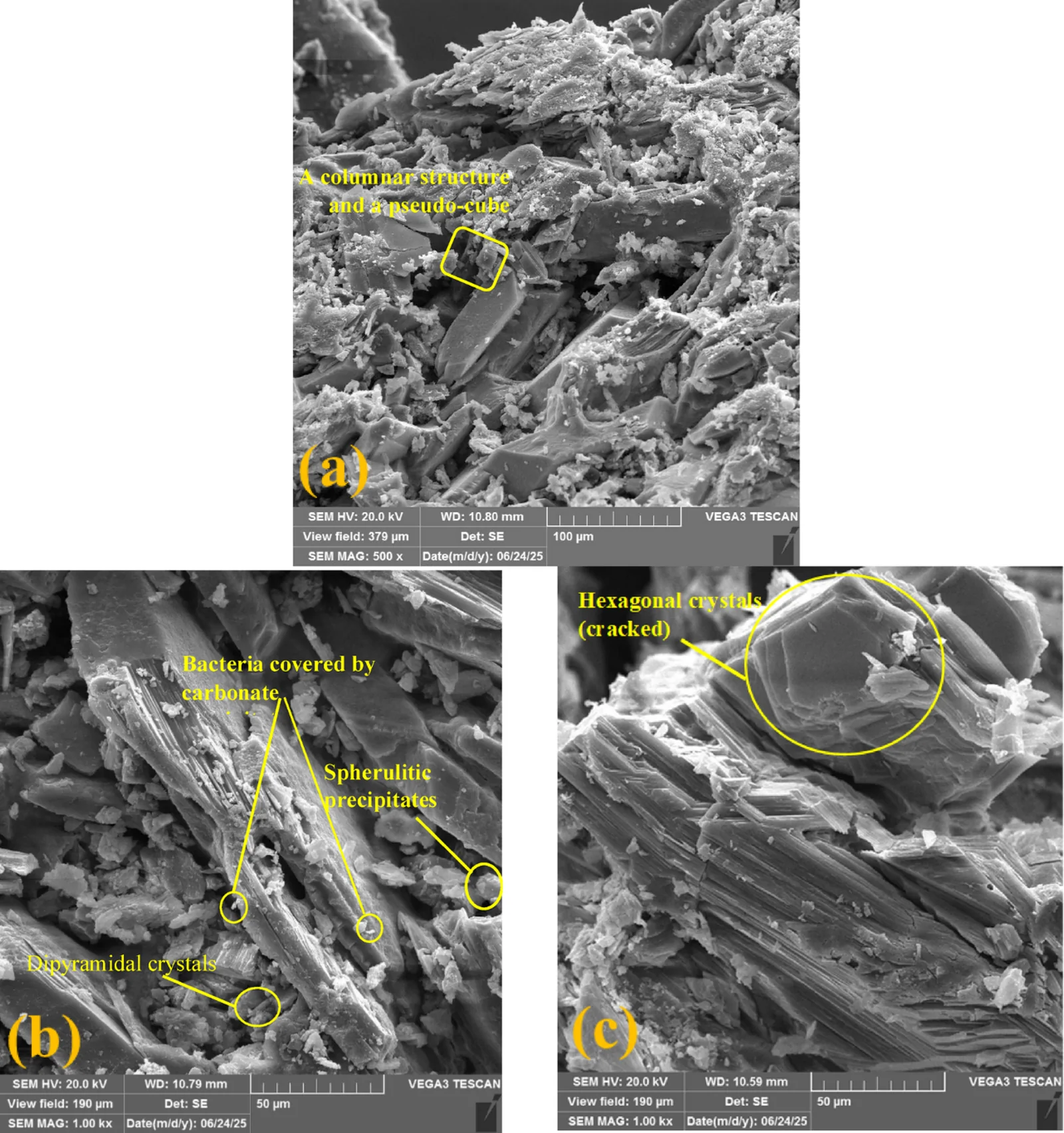
SEM micrographs of the biomineral precipitates capturing Pb, produced through microbially-facilitated carbonation: (**a**) Magnification 500 x: a columnar structure and a pseudo-cube can be detected indicating cerussite (**b**) Magnification 1000 x: Bacteria covered by precipitates, spherulitic (vaterite) crystals and bipyramidal (cerussite) crystals are noted. (**c**) Magnification 1000 x: Hexagonal crystals potentially indicating hydrocerussite are observed.

Figure 3 presents SEM micrographs of precipitates formed after lead fixation through microbially-facilitated carbonation. Bacteria covered with precipitates detected in Fig. 3 indicate a potential mechanism, where Pb removal can be facilitated through attraction of cations such as Pb^2+^ and Ca^2+^ to the bacterial cell surface, which has a negative charge. By reacting such cations with carbonate ions (formed after hydration of CO_2_ catalyzed by an anhydrase enzyme), minerals form and cover the bacterial surface. Spherulitic precipitates seen in Fig. 3 can be attributed to vaterite, a polymorph of CaCO_3_. Plate-like Hexagonal and columnar (forms such as dipyramidal) crystals point to the presence of lead carbonates such as hydrocerussite and cerussite, respectively. Such forms have been observed in previous studies^89,90^. Elemental mapping and energy dispersive analysis of precipitates (Figure S1, in online supplementary material) confirm the capture of carbon and fixation of lead in the form of calcium and lead carbonates. The rather unidirectional spatial distribution of Pb elements in elemental mapping points to an orthorhombic crystalline structure and potentially the higher amount of cerussite as compared to hydrocerussite precipitates.

**Fig. 4:**
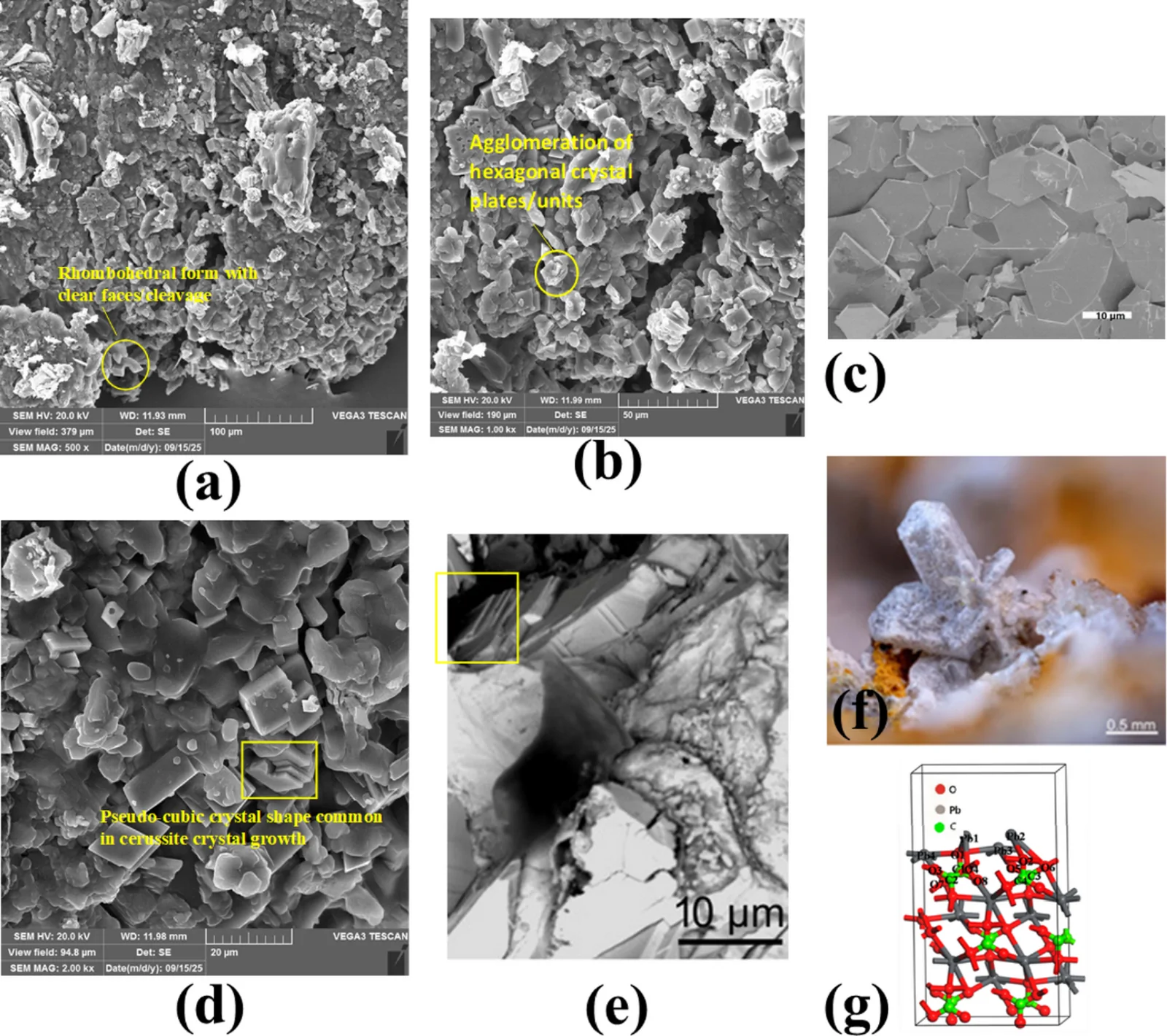
(**a**,**b**) SEM micrographs of the biomineral precipitates capturing Pb formed through enzymatically-facilitated carbonation (this study, magnification of 500x and 1kx), (**c**) SEM of pure hydrocerussite after Shi et al.^90^ under CC BY copyright license, (**d**) SEM image of precipitates obtained from enzymatically-facilitated carbonation for fixing lead (this study, magnification of 2kx), (**e**) SEM image of cerussite from Mastricarro Barite Mine (Catanzaro, southern Italy) after Bloise et al.^91^ under CC BY copyright license, (**f**) Optical image of cerussite from the same mine after Bloise et al.^91^, indicating columnar and planar growth tendency of cerussite following orthorhombic crystal structure, under CC BY copyright license, (**g**) Chemical structure of cerussite after Feng et al.^92^ under CC-BY copyright license.

Figure 4 presents the SEM micrographs of precipitates formed in enzymatically induced carbonation. The Rhombohedral forms with clear cleavage indicating calcite and pseudo-cubic crystal shape common in cerussite crystal growth^89–91^, as well as columnar structures indicating an orthorhombic crystal structure (either cerussite or aragonite) can be detected. Furthermore, elemental mapping and energy dispersive analysis results point to precipitation of both lead and calcium carbonates (Figure S2, in online supplementary material), the polymorphs of which are further (quantitatively) analyzed in the next sections. A visual comparison with the previously reported SEM micrographs of hydrocerussite and cerussite has also been included to better help recognize their presence in the SEM images. While hexagonal crystal shapes imply the presence of hydrocerussite, unidirectional growth and agglomerations in the form of columns or plates (forming a pseudo-cube) point to the presence of cerussite, which roots in its 3-D molecular structure illustrated in Fig. 4g.

**Fig. 5:**
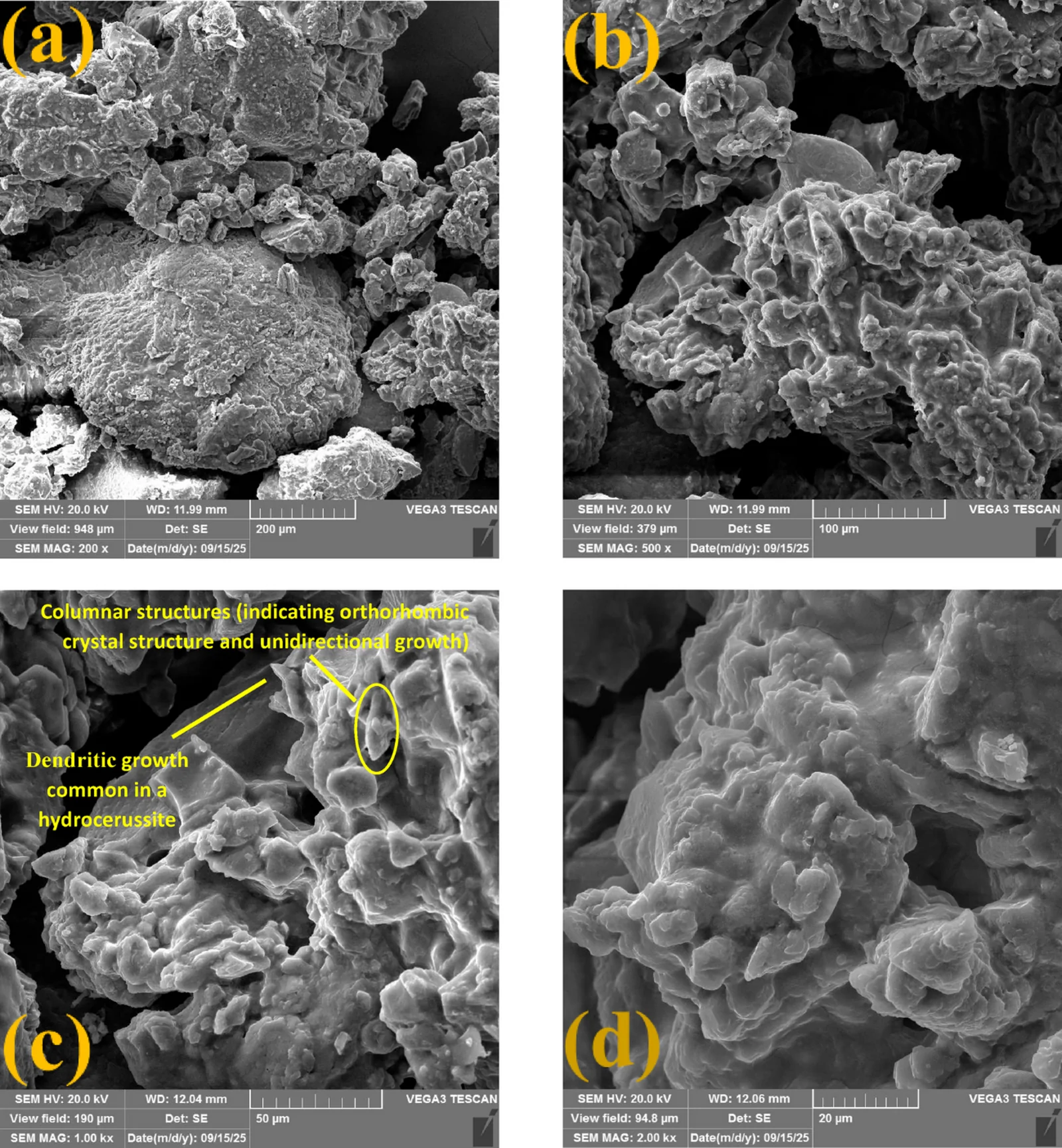
SEM micrographs of the biomineral precipitates capturing Pb produced through ureolytic biomineralization at magnification: (**a**) 200x, (**b**) 500x, (**c**) 1000x, (**d**) 2000x.

Figure 5 presents SEM micrographs of precipitates formed in ureolytic microbially induced carbonation and fixation of lead, where the unidirectional crystal growth and dendritic growth common in cerussite and hydrocerussite are observed. The same crystal structures have been reported in previous studies, indicating the formation of lead carbonate^89^. Figure S3 (supplementary material) confirms Pb fixation through the formation of lead carbonates and also their trapping within calcium carbonate minerals. FTIR, Raman, and XRD analyses presented in the next sections substantiate these mechanisms and bring further quantitative evidence.

#### Results of XRD and interpretation

**Fig. 6:**
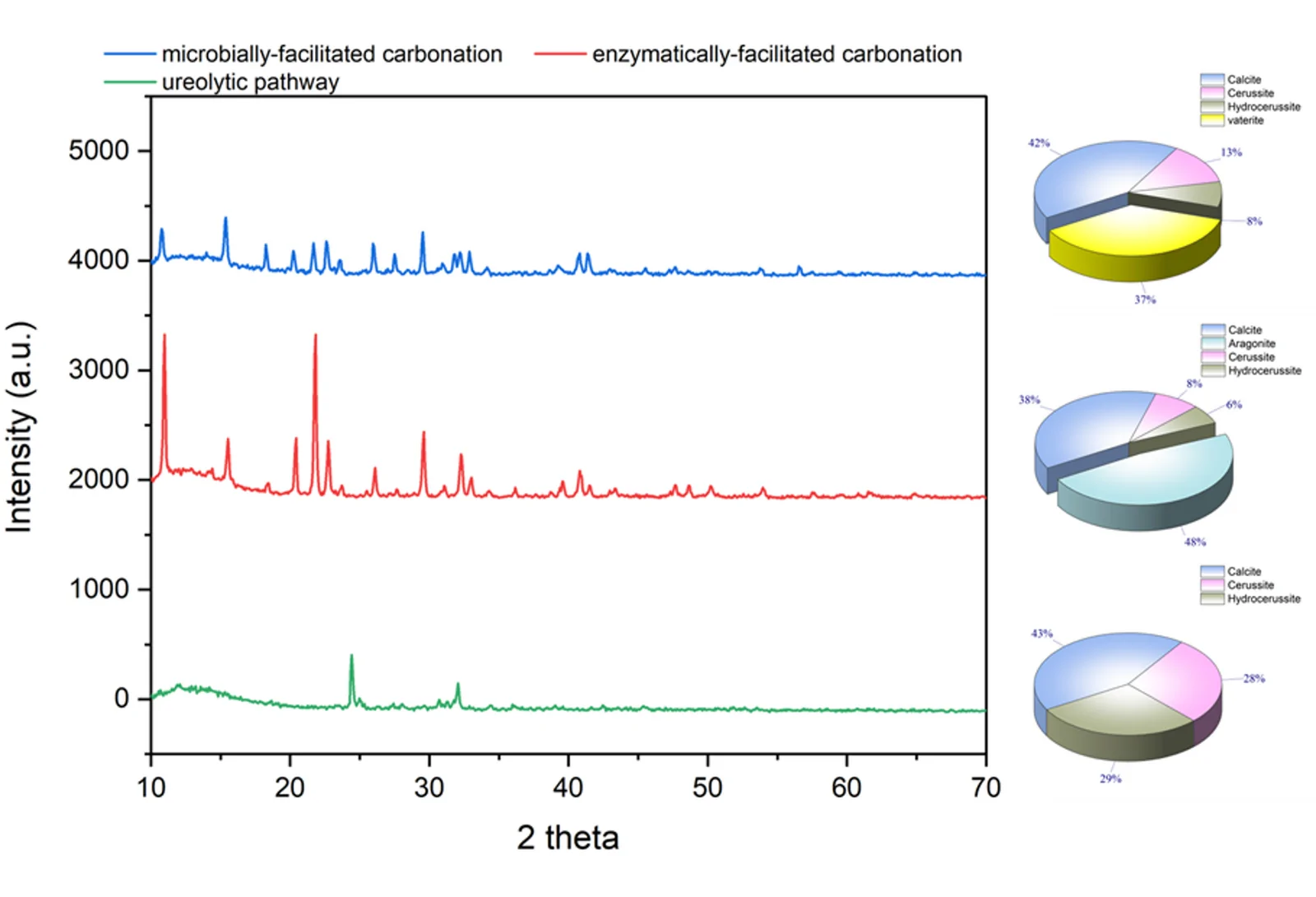
XRD spectrum of precipitates formed during lead fixation via microbially and enzymatically facilitated carbonation, and through the ureolytic pathway of microbially induced carbonate precipitation (MICP). The quantitative phase determinations are approximate and derived using Rietveld refinement.

Figure 6 presents the XRD micrograph as well as the percentage of different lead and calcium carbonate polymorphs in the precipitates obtained through lead fixation. XRD analysis of precipitates resulting from lead fixation through microbially-facilitated carbonation confirms the presence of multiple carbonate phases. Diffraction peaks at ≈ 25.4°, 36°, 45° (indicative of cerussite^89,93^, PbCO_3_), together with peaks at ≈ 27°, 34°, and 40°, associated with hydrocerussite^89,93^(Pb₃(CO_3_)_2_(OH)_2_), were observed. Among calcium carbonate polymorphs, the characteristic calcite pattern (following peak lists from Kim et al^94^. and Hunnicutt^95^ with strong peaks at 29.5°, 39.3°, 43.0°, 47.4°, and 48.6° are noticeable. Furthermore, peaks at ≈ 24.8°, 26.8°, 32.8°, and 50° (consistent with vaterite^95^ are noted with slight shifts to the left. The vaterite peaks, especially the main reflection at 24.8° is shifted to lower angles (e.g., ≈ 24.4°), which is a clear signature of lead substitution into the vaterite structure, forming a (Ca, Pb)CO_3_ solid solution. The presence of lead trapped in crystals of calcium carbonate results in shifting the reflections to higher d-spacing values (i.e., lower 2θ values). Similar patterns have been reported in previous studies^96^. In total, approximately 21% of the composition belongs to lead carbonates. Hence, both direct formation of lead carbonate and lead trapping within calcium carbonate crystals have contributed to lead fixation.

In contrast, the precipitates obtained through enzymatically facilitated carbonation exhibited a diffraction pattern dominated by calcite, with strong peaks observed at 29.5°, 39.3°, 43.0°, 47.4°, and 48.6°. Concurrently, distinct peaks are observed at ≈ 27°, 34°, and 40°, matching hydrocerussite. Additional peaks at ≈ 25.4°, 36°, 45° (specifying cerussite^89,93^ and at ≈ 27.2°, 33.1°, 45.8° (expected positions for aragonite^95,97^ are noted. In total, about 14% of the composition belongs to lead carbonate polymorphs; both direct formation of lead carbonate and lead trapping within calcite and aragonite crystals have contributed to lead fixation. In this method, calcite has also been the major polymorph among lead and calcium carbonates that have been formed.

Additionally, the precipitates obtained through ureolytic MICP exhibited diffraction peaks consistent with cerussite (≈ 25.4°, 36°, 45°) and hydrocerussite (≈ 27°, 34°, 40°), overshadowing peaks of calcite in intensity (with its distinct peaks recognized at ≈ 47.4°, 48.6°, 57.3°) are observed, indicating a higher percentage of lead carbonates as compared to calcium carbonates. In total, around 57.6% of the composition belongs to lead carbonate polymorphs; therefore, both direct formation of lead carbonate and lead trapping within calcite crystals have contributed to lead fixation. However, in this technique, a higher amount of lead has directly reacted with carbonate ions (generated by urea hydrolysis), and the rest of the lead cations present in the medium have been retained within calcite crystals.

The presence of thermodynamically less favorable polymorphs of vaterite and aragonite in precipitates obtained in microbially and enzymatically carbonation points to quick depletion of carbonate ions (following their reaction with available and abundant calcium cations^98^. The generated carbonate crystals trap lead within precipitates, but a lesser amount of lead carbonates forms. On the other hand, urea hydrolysis tends to raise pH (Fig. 2), and provides a rapid and continued supply of carbonates, allowing the formation of lead carbonates in higher amounts. No major preference in the formation of cerussite or hydrocerussite is seen in the latter technique (i.e., ureolytic MICP). Almost equal percentage of both lead carbonate polymorphs is noted in the precipitates, whereas a slightly higher amount of cerussite is detected among precipitates obtained through lead fixation using enzymatically- and microbially-induced carbonation (particularly in microbially-induced carbonation).

#### Results of FTIR

**Fig. 7:**
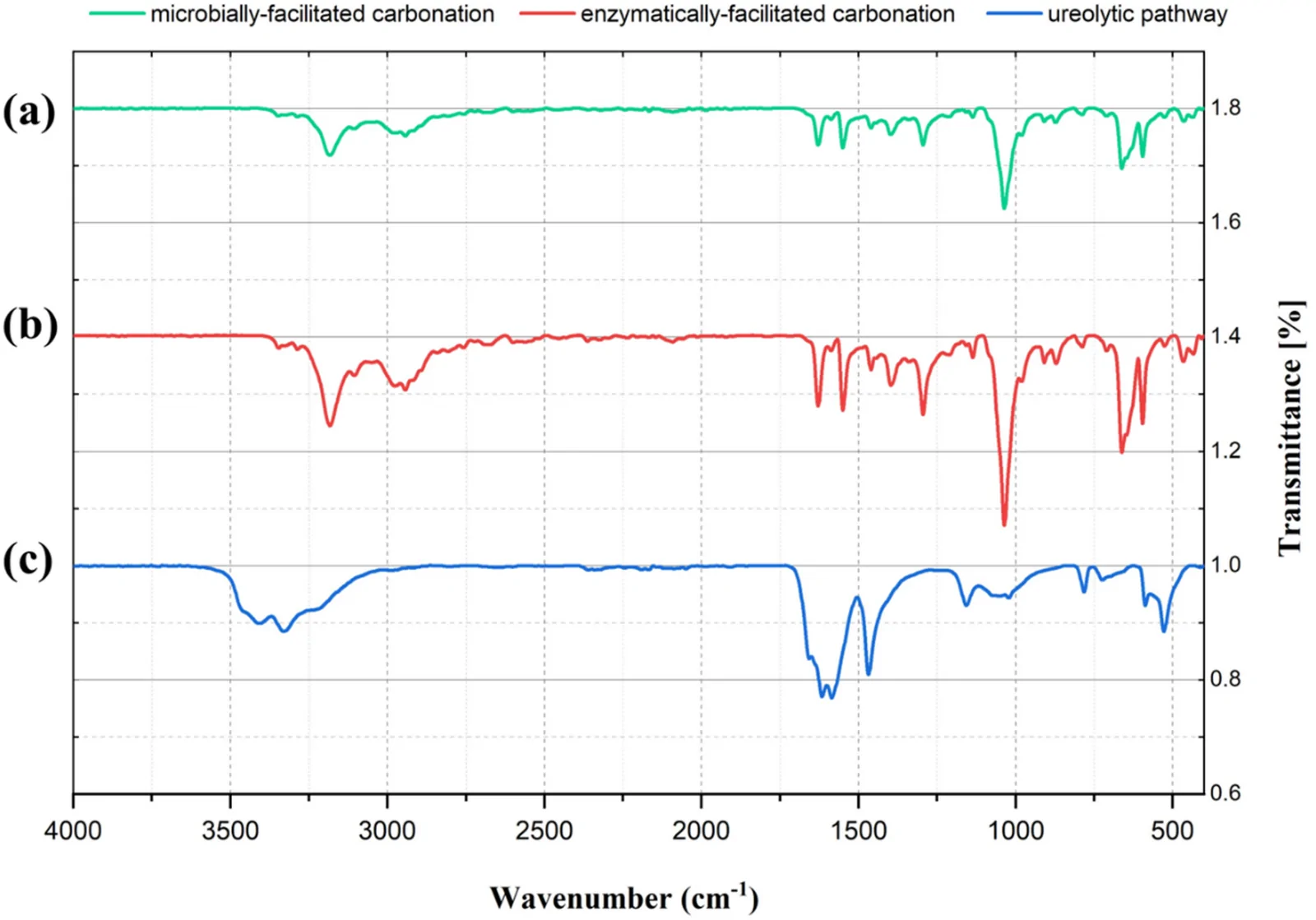
FTIR pattern of precipitates resulting from lead fixation through (**a**) microbially-facilitated carbonation, (**b**) enzymatically-facilitated carbonation, and (**c**) the ureolytic pathway of microbially-induced carbonate precipitation.

Figures 7a–c present FTIR results for precipitates containing lead in three methods of microbially-facilitated and enzymatically facilitated carbonation, together with ureolytic MICP. Peaks in the range 3200 to 3500 cm^− 1^ wavenumbers, which are indicators of O-H stretches, are the clearest indicator of hydrocerussite^99–102^. While these peaks are split and sparse in the microbially-facilitated carbonation and enzymatically-induced carbonation FTIR spectra (Fig. 7a,b), they are further intensified and appear as a doublet in the ureolytic microbially-induced carbonation (Fig. 7c), indicating a higher percentage of hydrocerussite crystals. This is in accordance with XRD analysis, indicating its increase from less than 10% in microbially-facilitated carbonation and enzymatically-facilitated carbonation to around 30% in ureolytic MICP fixing lead. Besides, peaks in the range of 1400 to 1500 cm^− 1^ (a pointer to asymmetric in-plane stretch, i.e., ν_3_ vibration modes of carbonate^103^ also appear in all three cases, confirming formation of carbonates (lead and calcium carbonates). In Fig. 7a,b and a triplet [596 cm^− 1^, 661 cm^− 1^, 710 cm^− 1^] is noticeable, where evolving lead and calcium carbonate phases can be recognized: the distinct ν_4_ bands of calcite (710 cm^− 1^)^103^, and bending vibration of OH (661 cm^− 1^)^104^ are visible alongside an emerging lead phase (596 cm^− 1^). Peaks in the 550–600 cm^− 1^ range are fingerprints for lead-oxygen (Pb-O) vibrations^104–106^. The slight shift of wavenumbers associated with calcite (712 cm^− 1^) to a lower value (710 cm^− 1^) is an indication of impurity and lattice distortion in calcite precipitates, potentially due to the presence of cations with a different mass^107^. These observations substantiate trapping of Pb elements within precipitated calcium carbonates in microbially-facilitated and enzymatically-facilitated carbonation techniques. In Fig. 7c, the peaks in 550–600 cm^−1^ range are much more pronounced and a clear doublet at [587 cm^− 1^, 527 cm^− 1^] is formed, signifying a higher percentage of cerussite in the ureolytic MICP method as compared to microbially- and enzymatically-facilitated carbonation approaches, which directly employ CO_2_ supply and rely on the CA-enzyme to facilitate carbonation (rather than using the urea hydrolysis).

#### Results of Raman spectroscopy

**Fig. 8:**
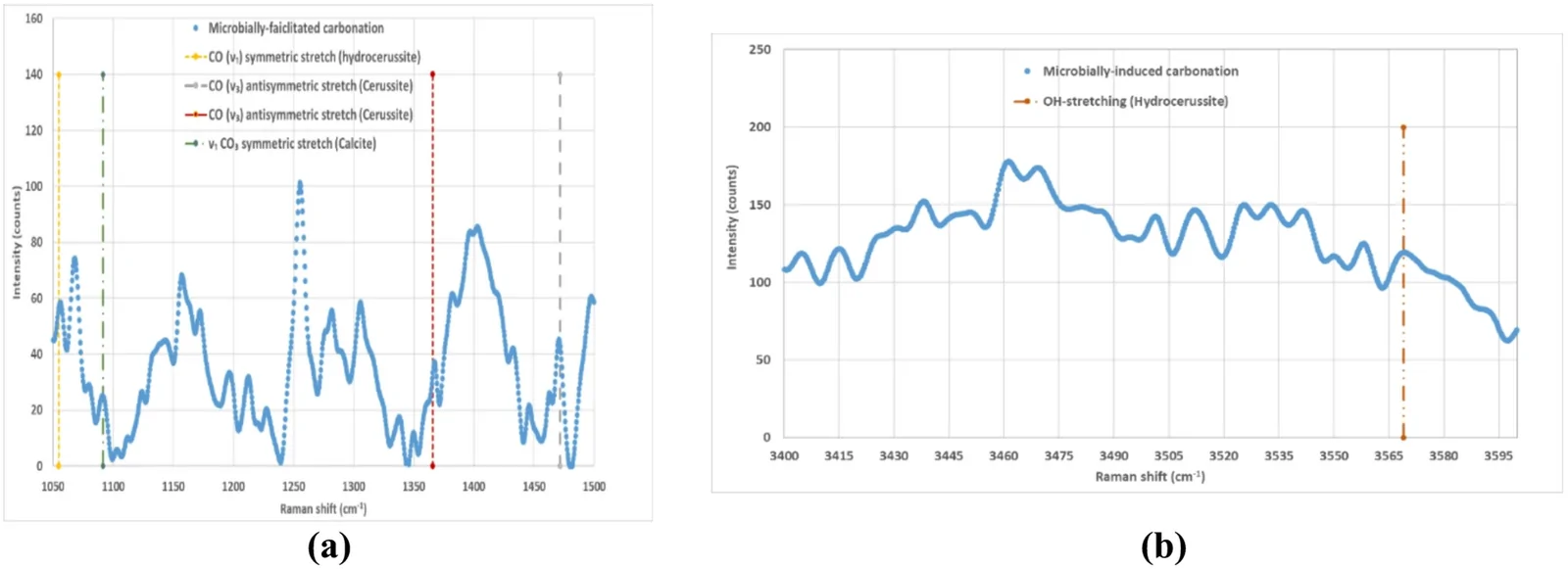
Raman shifts of precipitates resulting from lead fixation through microbially-facilitated carbonation: (**a**) 1000 to 1500 cm^− 1^, (**b**) 3400 to 3600 cm^− 1^.

**Fig. 9:**
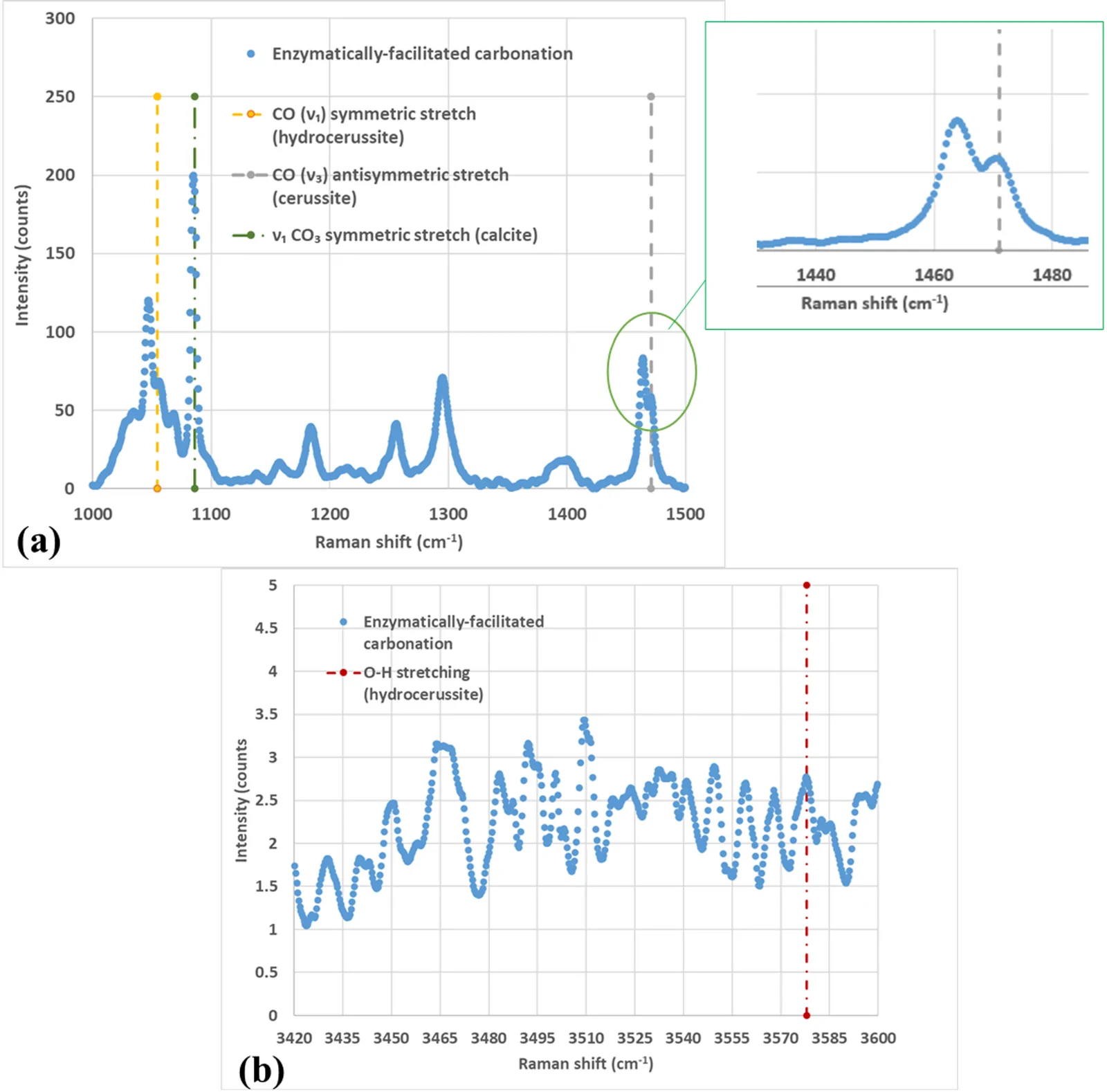
Raman shifts of precipitates resulting from lead fixation through enzymatically-facilitated carbonation: (**a**) 1000 to 1500 cm^− 1^, (**b**) 3400–3600 cm^− 1^.

As can be seen in Fig. 8a, peaks in the 1000 to 1100 cm^− 1^ range are indicative of the main vibration modes of carbonates^108,109^. These peaks appear close to the Raman shift values reported in the literature for calcite, cerussite, and hydrocerussite. For instance, CO (ν_1_) symmetric stretch for hydrocerussite (1054 cm^− 1^), CO (ν_3_) antisymmetric stretch for cerussite (~ 1473 and 1365) are present alongside a peak in the range 1086–1092 cm^− 1^ (indicative of ν_1_ CO_3_ stretch), notable for calcium carbonate polymorphs such as calcite^110–114^. Furthermore, the spectrum in 3400 to 3600 cm^− 1^ (Fig. 8b) shows peaks indicating OH-stretching^108,109^(notably, the peaks close to 3576 cm^− 1^ specify hydrocerussite). Figure 9a,b follow the same patterns, confirming the presence of cerussite, hydrocerussite, and calcite. However, the peak at 1086 is intense, clearly confirming the presence of calcite and well-formed calcite crystals despite their biogenic nature.

As revealed in Fig. 9b, a less intense peak close to 3576 cm^− 1^ points to a lower amount of hydrocerussite in enzymatically-facilitated carbonation as compared to microbially-induced carbonation, which is in agreement with XRD results (namely 6% in the former as compared to 8% in the latter). These observations confirm that the primary phase in the precipitates in the enzymatically-induced carbonation is calcite, in which Pb is captured and trapped, as evidenced earlier by energy dispersive analysis results.

**Fig. 10:**
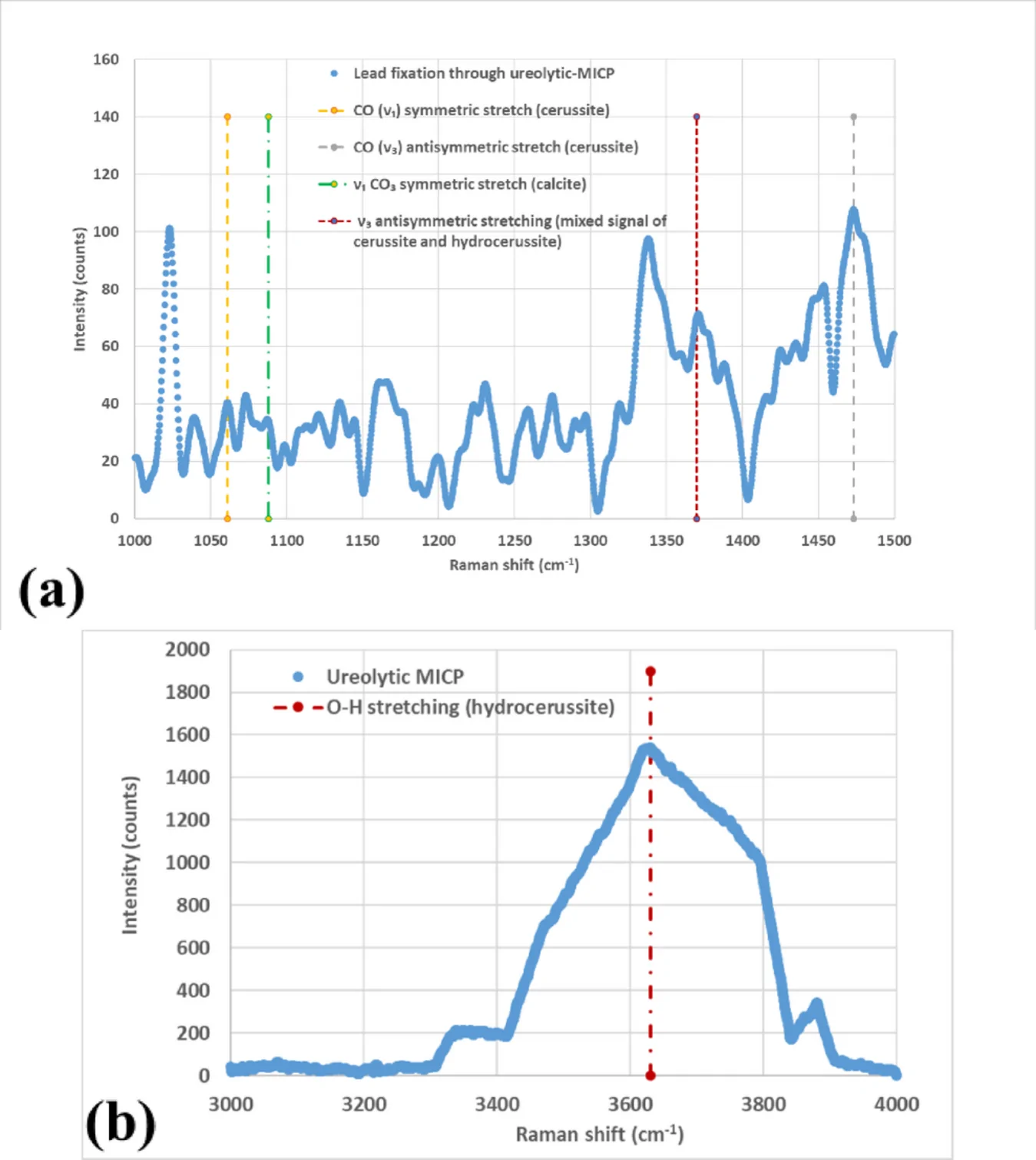
Raman shifts of precipitates resulting from lead fixation through the ureolytic pathway of microbially-induced carbonate precipitation: (**a**) 1000–1500 cm^− 1^, (**b**) 3000 to 4000 cm^− 1^.

The peaks confirming the presence of cerussite, hydrocerussite, and calcium carbonate (mainly calcite) are also noticeable in Fig. 10a,b, particularly the O-H stretching appears at a higher wave number 3630 cm^− 1^ and is markedly intense. As this peak is broadened, it seems to be formed as a mixed signal from hydroxyl groups in hydrocerussite as well as other compounds such as Ca (OH)_2_, potentially formed as by-products within lead carbonates in the presence of calcium cations. Besides, Fig. 10a points to a higher amount of cerussite and hydrocerussite, as the CO (ν_3_) antisymmetric stretch for cerussite and hydrocerussite is more pronounced, and the CO (ν_1_) symmetric stretch for cerussite (at 1061 cm^− 1^)^108^ is detected, confirming XRD results. The results of microfabric analysis inclusive of SEM, EDX, XRD, FTIR, and Raman all confirm Pb removal and its mineral trapping both within carbonate crystals and by precipitating as lead carbonates; although the former mechanism is the major mechanism in microbially- and enzymatically-facilitated carbonation and the latter is more pronounced in ureolytic-MICP, potentially due to difference in its kinetics as compared to ca-facilitated carbonation, which results primarily in calcium carbonate crystals as compared to lead carbonates.

## Comparison of biomineralization and removal mechanisms in ureolytic MICP versus bacterially and enzymatically-facilitated carbonation and biomineralization: discussion of the results

FTIR, Raman spectroscopy, and X-ray diffraction analysis pointed to the formation of lead and calcium carbonates. Both the formation of lead carbonate as a separate phase as well as lead incorporation in the calcium carbonate crystal lattice and surface adsorption of lead cations were substantiated by the results. However, it was observed that the major lead removal mechanism in the ureolytic MICP is the formation of lead carbonate as a separate phase (i.e., as the higher percentage of lead carbonate was observed among the precipitates) whereas in the microbially- and enzymatically-facilitated carbonation and biomineralization, the percentage of calcium carbonate polymorphs was higher and the incorporation of lead cations into the lattice of calcium carbonate crystals and on their surface was evidenced by the spectroscopy results as well as SEM and EDS analyses.

The different outcomes stem from the fundamental differences in how the two enzymatic systems (CA versus urease) deliver and manage the key reactants—specifically carbonate ions (CO_3_^2−^) and pH—and how these conditions favor either direct lead precipitation or lead incorporation into calcium carbonates. The process is largely governed by reaction kinetics, the thermodynamics of mineral formation, and the dynamic competition between Pb^2+^ and Ca^2+^ for available carbonate. These governing factors are discussed hereafter:

### The role of CO_2_: slow hydration vs. rapid supply

The central difference lies in how carbonate ions are generated:

- **CA-facilitated carbonation technique (i.e.**,** Direct CO**_**2**_ **Injection)**: The CA enzyme catalyzes the reaction: CO_2_ + H_2_O → H^+^ + HCO_3_^-^. This reaction does not directly produce carbonate ions but rather bicarbonate (HCO_3_^-^) and a proton (H^+^). This tends to create a neutral or even slightly acidic local environment around the enzyme. The formation of carbonates then depends on a secondary equilibrium (HCO_3_^-^ ⇌ H^+^ + CO_3_^2-^), which is limited in neutral to acidic conditions. This results in a relatively low, steady-state flux of carbonate ions.
- **Ureolytic MICP (Urea Hydrolysis)**: The urease enzyme breaks down urea (i.e., CO(NH_2_)_2_ + 2H_2_O → 2NH_4_^+^+ CO_3_^2-^). This is a much more direct pathway, which also co-produces ammonium (NH_4_^+^), raising the pH. The high pH is crucial as it immediately creates an environment highly favorable for carbonate precipitation. Therefore, ureolytic pathway provides a rapid, high-flux “burst” of both carbonate ions and alkaline conditions, which is a key factor favoring the direct formation of lead carbonate minerals.

### Kinetics of precipitation: competition of lead vs. calcium for available carbonates

In both approaches, the ions are in direct competition to react with the available carbonates. However, the competition is controlled by the speed of carbonate formation discussed in the previous section, together with reaction kinetics and abundance of cations.

- **In Ureolytic MICP**: The extremely high flux of carbonates can exceed the capacity of Ca^2+^ cations to react immediately, providing Pb^2+^ cations the opportunity to precipitate directly as lead carbonates^,^ making them the dominant removal mechanisms.
- **In CA-facilitated carbonation technique**: The formation of CO_3_^2-^ is limited. Therefore, the abundance of Ca^2+^ cations as compared to Pb^2+^ drives the reaction toward higher precipitation of calcium carbonate. Despite the formation of lead carbonates in smaller amounts as compared to ureolytic MICP, it is not the primary removal mechanism. As the calcium carbonate (CaCO_3_) begins to precipitate slowly, Pb^2+^ ions in the solution are removed through other removal mechanisms, which are:
- **Adsorption**: Pb^2+^ ions physically adhere to the surface of the growing CaCO_3_ crystals.
- **Co-precipitation**: A number of Pb^2+^ ions are also incorporated into the CaCO_3_ crystal lattice as it forms.

**Fig. 11:**
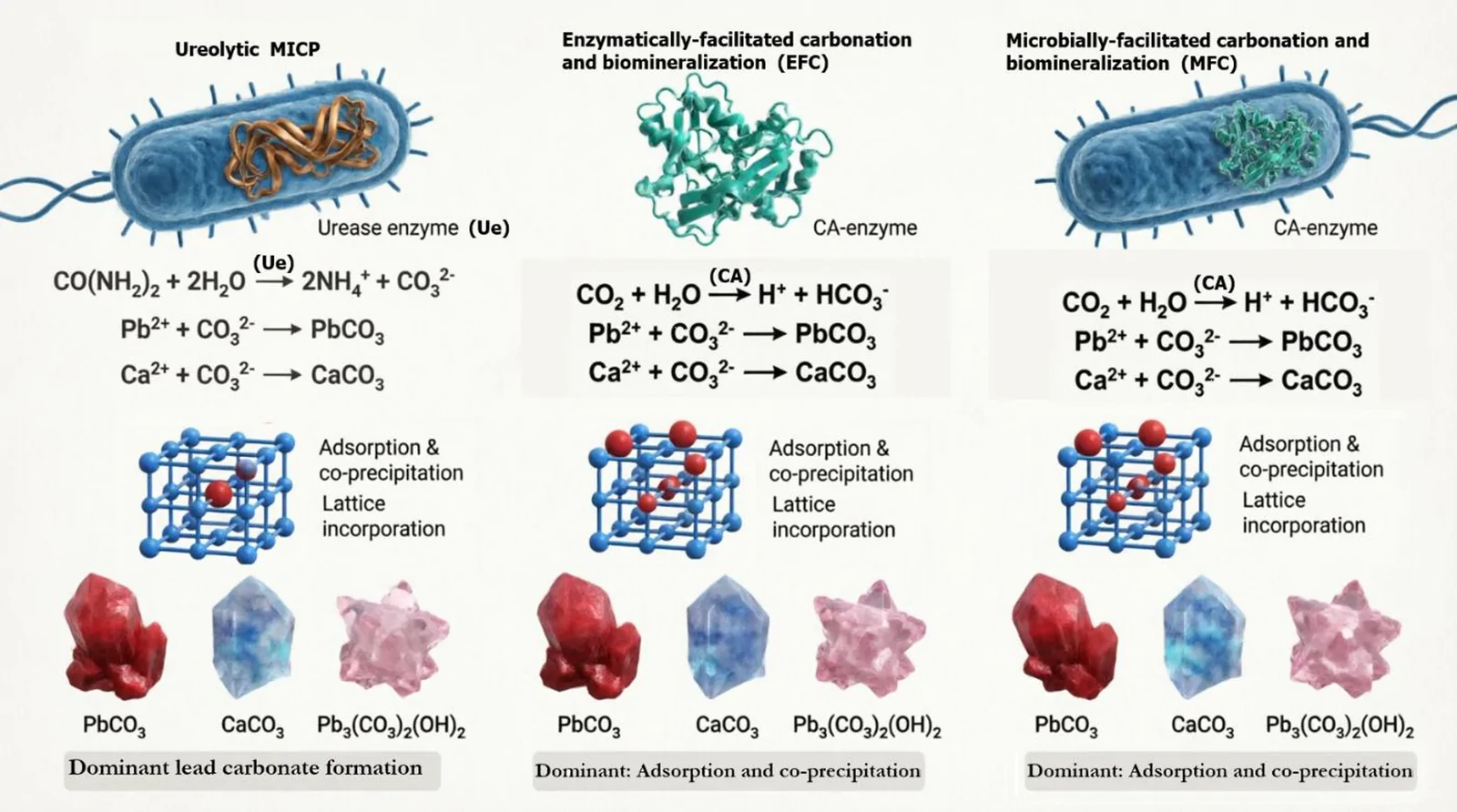
Lead fixation via Ureolytic MICP, EFC and MFC: summary of products and removal mechanisms.

Figure 11 schematically presents and summarizes the involved chemical reactions and dominant mechanisms.

## Heavy metal fixation through biologically facilitated carbonation (BFC) versus ureolytic MICP: advantages, disadvantages, limitations, and future outlook

Table 2 summarizes and compares the results of this study with some major studies previously reported in the literature on the use of MICP and EICP for heavy metal removal.

**Table 2:** Comparing Pb removal efficiency of the results from this study and those of the literature.

No.	Soil or aqueous solution	Method	Heavy metal type	Heavy metal concentration	Bacterial concentration in terms of OD_600_	Enzyme type/concentration/activity	Removal efficiency	Ref.
1	Aqueous solution	Ureolytic MICP	Pb	Aqueous solution (0.5 mM)	*Sporosarcina pasteurii*/ OD = 1.6	–	98%	This study
2		MFC (with Tris buffer)	Pb		*Sporosarcina pasteurii*/ OD = 1.6	–	99%	
3		EFC (with Tris buffer)	Pb		–	Carbonic anhydrase (3 mg L^− 1^)	99%	
4		MFC (without Tris buffer)	Pb		*Sporosarcina pasteurii*/ OD = 1.6	–	95%	
5		EFC (without Tris buffer)	Pb		–	Carbonic anhydrase (3 mg L^− 1^)	78%	
6		Ureolytic MICP	Zn, Cd, Ni	Aqueous solution (4 mM) & (6 mM)	*Bacillus subtilis HMZC1 (B2)*,* Bacillus sp.* *HMZCSW (B6)*,* Comamonas sp. HMZC (B11)/* OD = 1.0	–	All three strains achieved > 93% removal of Zn^2+^, Ni^2+^, and Cd^2+^ within 72 h.	Rajasekar et al^115^.
7		Ureolytic MICP and Ureolytic EICP	Pb, Cu	Solution (5–50 mM) Pb^2+^ concentration	*Sporosarcina pasteurii*/OD = 1.8–2.1	Urease from *Canavalia ensiformis/342.7 U/g*	~ 100% (Pb removal in range 5–50 mM: for both MICP and EICP); >20% (Cu removal in a range 5–50 mM under EICP); >77% (Cu removal in range 40–50 mM under MICP)	Wang et al.^116^
8		Ureolytic MICP	Pb, Cd, Cu	Solution (50 to 800 mg/L)	*Sporosarcina pasteurii/OD = 0.4*	–	In the presence of a calcium source, higher removal efficiency: Cd^2+^ (98.0–99.0%) Cu^2+^ (78.1–82.1%) and Pb^2+^ (98.0–100.0%)	Li et al.^117^
9		Ureolytic EICP	Cd, Pb, Cr	Solution (50–500 mg/L)	–	Soybean-derived urease/0.5–2.0 U/mL (with an optimum of 1.0 U/mL)	> 70% heavy metal immobilization (> 80%: Cd, and Pb; >75% Cr, in aqueous solutions of a single metal).	Xu et al.^118^
10		Ureolytic EICP	Pb	Solution (5–50 mM)	*Sporosarcina pasteurii/OD = 1*	*20% (v/v) bacterial enzyme extract solution/5–7 U/mg*	Up to 38.1% (achieved for 20 mM Pb concentration after 20 h of incubation)	Joukar et al.^119^
11	Contaminated soil	Ureaolytic EICP	Pb	Contaminated soil (2.7 mg kg^−1^)	–	Ureases extracted from soybean (SCU), black-bean (BCU), sword-bean (SWCU) and watermelon-seed urease (WCU) (20–120 g L^− 1^)	SWCU achieved the highest exchangeable-Pb removal 94.11%, compared to WCU (88.1%), BCU (86.52%), SCU (85.12%)	Bian et al.^120^
12		Ureolytic MICP with and without graphene	Pb	Contaminated loess soil (500 mg kg^− 1^)	*Sporosarcina pasteurii*/OD = 1.70	–	~ 39.6% reduction in Pb leaching (MICP alone); up to ~ 61.6% with graphene oxide (GO)	Wang et al.^121^

As can be seen, these studies rely mostly on the ureolytic pathway, namely the hydrolysis of urea to produce carbonate ions needed for lead and calcium carbonate precipitation. However, the ureolytic pathway results in the production of ammonium ions, which are not environmentally desirable, particularly when provided at large volumes. Furthermore, as discussed by Wang et al.^116^, ammonium production can lead to metal-ammonia complexes (such as copper-ammonia complex with the chemical formula of [Cu (NH_3_)_4_(H_2_O)_2_]^2+^, bringing metal cations into a free state, reducing the remediation efficiency. This has been particularly suggested for Cu^2+^ by Wang et al.^116^. Their observations can potentially explain the generally lower removal efficiencies observed for copper as compared with lead, cadmium, zinc, nickel, and chromium (Table 2). Therefore, the ammonium-free nature of the suggested method in this study, namely microbially-facilitated carbonation (MFC) and enzymatically-facilitated carbonation (EFC), is an important advantage, alleviating the mentioned drawbacks of ureolytic MICP and ureolytic EICP, while bringing about similar removal efficiencies. It is notable that the current study has focused on lead removal through MFC and EFC, and its potential for the fixation of other heavy metals is yet to be examined in future studies. Furthermore, this method provides a method to directly capture CO_2_ through forming lead and calcium carbonates rather than relying on the hydrolysis of urea. In the current study, enzymatically-facilitated carbonation was found to be influenced by the presence or absence of Tris Buffer, which was attributed to the potential sensitivity of the enzymatic method to pH variation during the lead removal process and precipitation formation. On the contrary, microbially-facilitated carbonation was found to be more resilient.

When comparing previous studies, it seems that ureolytic MICP has shown a superior performance in aqueous solutions as compared to contaminated soils (i.e., particularly when ureolytic MICP and EICP performance in contaminated soils are compared). In some studies, the MICP removal efficiency in soils has been augmented by an additive such as graphene oxide^121^. In fine-grained soils, particularly, the efficiency of microbially-induced biomineral precipitation and heavy metal fixation is limited due to soil throat sizes and the restriction of bacterial mobility, transport, and eventually activity. Although the present study has mainly focused on aqueous solutions and not soil, the same restrictions can particularly apply to the MFC method. Therefore, the performance of this method in contaminated soils of different grain and pore sizes has to be studied in future studies. Furthermore, as the proposed techniques in this study for heavy metal fixation rely on direct CO_2_ supply, the CO_2_ diffusion should be enhanced within soil with particular and tailored methods, such as dedicated channels or perforated pipes, which need specific consideration and tailored design.

It is noteworthy that the presented study considered a fixed Pb concentration to be of relevance for most low to moderately-contaminated real-world cases presented in Table 1. The studies presented in Table 2 indicate that a Pb concentration increase up to 50 mM has not reduced the removal efficiency of biologically-induced precipitation for Pb removal in Aqueous solutions. Furthermore, high dosages as high as 30 mM Pb concentration have been proposed to be of little toxicity and marginal effect on bacterial growth and associated urease activity of strains such as *Sporosarcina pasteurii*^122^. Despite these findings, as the presented study relies on a different pathway, namely, hydration of CO_2_ through carbonic anhydrase, it is suggested to perform studies with higher Pb concentration to evaluate the effect of Pb concentration on the efficiency of the method for the cases of extremely contaminated industrial streams.

As discussed earlier, MFC and EFC use direct supply of CO_2_, and therefore, do not result in production of ammonium ions, in contrast with ureolytic MICP. However, it is notable that there are methods proposed in the literature to alleviate the problem of ammonium production in ureolytic MICP. For instance, as reported by Lee et al.^123^, appropriate post-treatment rinsing techniques can effectively remove virtually all ammonium produced by ureolytic MICP from up to 5 cubic ft of bio-cemented soil. Furthermore, Lee et al. approach enriches native ureolytic bacteria, thereby avoiding the costs to cultivate and transport large volumes of culture to a remediation site. Inspired by the work of Lee et al.^123^, one can suggest enriching native bacteria capable of producing carbonic anhydrase, combined with the use of direct CO_2_ supply for lead fixation, to be examined in future studies for remediating contaminated soils. The use of bacterial powders^55^ consisting of CA-producing bacterial spores, and their on-site activation, can also be studied.

A detailed assessment of the environmental sustainability of EFC, MFC and ureolytic MICP necessitates comparing the carbon and energy footprint of the rinsing methods^123^ versus the footprints of producing and transporting large volumes of culture to an MFC treatment site or of partially purifying CA and transporting that to an EFC site. Moreover, to evaluate the environmental sustainability of ureolytic MICP, EFC and MFC, a “use case” and target performance criteria, as well as (2) pilot experiments, defining the level and energy costs, are required to be defined and assessed. If the “use case” is remediating soil, colloidal filtration restricting useful pumping distances for MFC suspensions have also to be considered, as discussed by DeJong et al.^124^.

As this study aimed to examine the feasibility and potential of microbially-facilitated carbonation (MFC) and enzymatically-facilitated carbonation (EFC) and their induced biomineralisation for lead fixation and removal, the current study considered minimum environmental complexity to ensure reproducibility of results and controlled experimental conditions enabling a fair comparison among EFC, MFC and well-established ureolytic MICP. This study, therefore, focused on single-metal aqueous solutions and short-term experiments. Future studies are, hence, encouraged to investigate competing ions and complex environmental conditions such as variable pH. Furthermore, in this study, the use of sealed box for CO_2_ treatment limited our access to aqueous solutions during treatment to collect data over variation of pH and heavy metal concentrations with time. To investigate pH variation and metal concentration with time, tailored experiments are suggested to elucidate involved kinetics and mechanisms.

## Summary and concluding remarks

In this study, novel biological techniques, namely microbially-facilitated carbonation through *Sporosarcina pasteurii* (utilizing direct supply of CO_2_ and no urea addition) and enzymatically-facilitated carbonation through the use of cell-free bovine Carbonic Anhydrase (CA) enzyme, were utilized to fix lead in aqueous solutions. The major findings are as follows:

- The microbially-facilitated and enzymatically-facilitated carbonation showed promising results and up to 99% removal capacity of lead under the studied conditions, similar to ureolytic MICP (i.e., 98% in this study).
- Addition of Tris buffer markedly increased the efficiency of enzymatically-facilitated carbonation in fixing lead (i.e., from 78% to 99%), and increased the microbially-facilitated carbonation efficiency from 95% to 99%. Based on the values of initial and final pH of the treated solutions, the effect of Tris Buffer on the CA-enzyme on CO_2_ hydration potential was discussed. It was found that the addition of Tris Buffer greatly enhances the CA-enzyme CO_2_ hydration potential through its effects on the pH of solutions. However, the absence of reaction kinetics curves and leaching/long-term stability tests remains a significant challenge and limitation to further validate and understand the mechanisms behind the observed removal mechanisms as well as the long-term removal efficiency of EFC and MFC methods.
- Microfabric analysis (SEM, XRD, FTIR, and Raman analyses) of the precipitates obtained in each of these techniques revealed that lead and calcium carbonates, such as calcite, cerussite, and hydrocerussite, were present in the precipitates. Particularly, XRD analysis indicated that the composition of carbonates in precipitates is as follows: MFC: calcite (42%), vaterite (37%), cerussite (13%), EFC: calcite (38%), aragonite (48%), cerussite (8%) and hydrocerussite (6%), and in ureolytic MICP: calcite (43%), cerussite (28%) and hydrocerussite (29%). This means that in ureolytic MICP, a higher percentage of lead removal has been achieved through lead carbonate precipitation, while in MFC and EFC, the faster kinetics and CO_2_ hydration have led to primarily calcium carbonate precipitation, and lead cations have been attracted to the surfaces of calcium carbonates and also trapped within their lattice as the primary removal mechanisms.

The proposed mechanisms for Pb fixation in the introduced techniques are as follows:

- MFC and EFC methods rely on catalyzed hydration of carbon dioxide through cell-free or bacterially produced CA. This eventually results in the formation of carbonate ions. Alternatively, in ureolytic MICP, hydrolysis of urea using the urease enzyme produced by bacteria results in the generation of carbonate ions. The reaction of carbonate ions and Ca^2+^ and Pb^2+^ present in the medium eventually results in the precipitation of calcium and lead carbonates.
- Therefore, not only is Pb removed through the formation of lead carbonates but also trapped within the crystal lattice of calcium carbonates, likely substituted through isomorphic substitution due to the similar ionic radius of Ca^2+^ and Pb^2+^ (≈ 1.00 Å and ≈ 1.19 Å, respectively) and identical charge.
- Finally, as bacterial cells and calcium carbonate crystals possess negative surface charges, Pb immobilization also occurs through its attraction to their surface.

As this study has been mainly focused on investigating the feasibility of using enzymatically-facilitated carbonation and microbially-facilitated carbonation for lead removal, for the first time, there are several potential research areas that remain unexplored and are introduced hereafter:

- This study examined Pb removal in aqueous solutions (after 7 days of exposure to CO_2_). Long-term stability of Pb carbonates needs to be examined. The effect of different Pb concentrations and reagent concentrations can also be examined to find the optimum scenario for Pb removal through MFC and EFC.
- The efficiency of EFC and MFC techniques for Pb fixation in contaminated soils must be examined. Furthermore, field deployment and delivery strategies for enzymatic and bacterial solutions, and designed methods to enhance CO_2_ injection inside and diffusion through soil media have to be explored and identified.
- Hybrid approaches combining MFC/EFC with adsorptive materials and nanoparticles such as graphene and biochar, as well as biopolymers, are other potential research directions.
- This study utilized an all-in-one approach where bacterial solution and calcium source were added to the contaminated aqueous solution, and then it was exposed to CO_2_ gas. Another potential method to use EFC and MFC in industrial scale is: Phase (I) to expose a solution containing calcium source and bacterial/CA enzyme source to CO_2_, separate calcium carbonate crystals generated, and then Phase (II): to use these biologically synthesized Pb adsorptive material (calcium carbonate crystals) to fix lead or other contaminants in the contaminated land or stream. As the method does not require hydrolysis of urea and relies on direct biologically-facilitated CO_2_ sequestration, it brings dual environmental benefits. Although this two-stage method offers further controllability on the production of calcium carbonate crystals (by their generation a priori in a factory) and then simpler implementation in the field, the efficiency of such a two-stage technique has to be explored in future studies.

The effect of other calcium sources on the efficiency of EFC and MFC has to be examined, and their removal efficiency needs to be studied for other heavy metals such as cadmium and arsenic.

## Supplementary Information

Below is the link to the electronic supplementary material.

Supplementary Material 1

## Data Availability

The data that support the findings of this study are available from the corresponding author upon reasonable request.
