# Supplementary material for: Lead contamination fixation through CO2-based biomineralization
Source: Sci Rep. 2026 Jun 22;16:28568. doi: 10.1038/s41598-026-58559-y (PMC13572381; doi:10.1038/s41598-026-58559-y)
Supplement: Supplementary file 1 [file 41598_2026_58559_MOESM1_ESM.docx]

**Supplementary material**: the results of elemental mapping, and EDS analysis of precipitates

Figures **S1**, **S2** and **S3**, provided hereafter, present the results of elemental mapping, and EDS analysis of precipitates formed during lead removal through microbially-facilitated carbonation, enzymatically-facilitated carbonation and ureolytic MICP, respectively. For discussion of the results, please refer to the main text of the manuscript.

|  |  |
| --- | --- |
|  |  |
|  | |
| **Figure S1.** SEM, elemental mapping, and EDS analysis of precipitates formed by the microbially facilitated carbonation method for fixing lead; (a) SEM image of the precipitated matrix, (b) SEM image overlaid with combined elemental mapping, (c) Pb elemental mapping (red) showing a non-uniform spatial distribution, (d) Combined elemental map of Pb (red), C (yellow), Ca (blue), and O (green), and (e) EDS spectrum with quantitative elemental composition (wt%). | |

|  |  |
| --- | --- |
|  |  |
|  | |
| **Figure S2.** Elemental mapping and energy dispersive x-ray analysis of precipitates formed in enzymatically-facilitated carbonation method for fixing lead, (a) SEM image of the precipitated matrix, (b) SEM image overlaid with combined elemental mapping, (c) Pb elemental mapping (red) substantiating the formation of minerals, capturing lead, (d) Combined elemental map of Pb (red), C (green), Ca (cyan), and O (purple), and (e) EDS spectrum with quantitative elemental composition (wt%). | |

|  |  |
| --- | --- |
|  |  |
|  | |
| **Figure S3.** Elemental mapping and energy dispersive X-ray analysis of precipitates formed in the urelytic pathway for fixing lead: (a) SEM image of the precipitated matrix, (b) SEM image overlaid with the elemental mapping of precipitates (C= green; Ca=cyan; Pb=red). (c) Pb elemental mapping (red) indicating formation of minerals, containing lead, (d) Combined elemental map of Pb (red), C (green), Ca (cyan), and O (purple), and (e) EDS spectrum with quantitative elemental composition (wt%). | |
